# Novel Small Molecule DZ-865B Effectively Degrades BCL6, Promotes Apoptosis and Reduces Proliferation of Diffuse Large B-Cell Lymphoma Cells

**DOI:** 10.32604/or.2026.068695

**Published:** 2026-02-24

**Authors:** Yanfeng Wang, Xinyi Chen, Yichen Yin, Tao Li, Jing Chen

**Affiliations:** 1 School of Basic Medical Sciences, Ningxia Medical University, Yinchuan, China; 2 Key Laboratory of Fertility Maintenance Ministry of Education, Ningxia Medical University, Yinchuan, China; 3 Department of Oncology, General Hospital of the Ningxia Medical University, Yinchuan, China

**Keywords:** Diffuse large B-cell lymphoma (DLBCL), B-cell lymphoma 6 (BCL6), proteolysis-targeting chimeras (PROTACs), proliferation

## Abstract

**Objectives:**

B-cell lymphoma 6 (BCL6) is a transcriptional repressor whose overexpression is closely linked to the progression of diffuse large B-cell lymphoma (DLBCL), making it a promising therapeutic target. This study aims to identify a novel small molecule, synthesized via proteolysis-targeting chimeras (PROTACs), capable of degrading BCL6, thereby inhibiting DLBCL growth and providing a foundation for future preclinical studies.

**Methods:**

The expression of BCL6 in DLBCL was analyzed using The Cancer Genome Atlas (TCGA) database and the Human Protein Atlas. Western blotting assays confirmed BCL6 expression in tumor cell lines, leading to the identification of the small molecule compound DZ-865B. To evaluate DZ-865B’s *in vitro* efficacy, multiple assays were performed, including protein immunoblotting, immunofluorescence, reverse transcription quantitative PCR, EDU proliferation, and soft agar cloning assays.

**Results:**

TCGA analysis revealed significant overexpression of BCL6 in DLBCL (*p* < 0.05), corroborated by immunohistological staining and western blotting. DZ-865B induced BCL6 degradation in DLBCL cell lines (OCI-LY-1 and SU-DHL-4) in a concentration- and time-dependent manner, and induced the degradation of nuclear BCL6 through the ubiquitin-proteasome pathway. Notably, DZ-865B did not alter BCL6 mRNA levels but modulated downstream gene expression, leading to the activation of apoptosis pathway proteins and inhibition of DNA synthesis, effectively suppressing DLBCL cell growth.

**Conclusion:**

This study demonstrates that the small molecule DZ-865B targets and degrades BCL6 in DLBCL cells, promoting apoptosis and inhibiting cellular proliferation. These findings highlight DZ-865B as a potential therapeutic agent for diffuse large B-cell lymphoma.

## 1 Introduction

Recent GLOBOCAN data highlight the global burden of non-Hodgkin lymphoma (NHL), with approximately 553,000 new cases anticipated in 2022, accounting for 5.5% of all new cancer diagnoses. Additionally, NHL is projected to cause around 250,000 deaths worldwide, representing 2.4% of all cancer-related mortality [1]. Diffuse large B-cell lymphoma (DLBCL), the most common subtype of NHL, comprises roughly one-third of all NHL cases [2–5]. Current treatment for DLBCL primarily relies on chemoimmunotherapy, yet approximately 30% of patients experience early relapse, and 10% display treatment-refractory disease [6]. The germinal center B-cell (GCB-) subtype, defined by Cluster of Differentiation 10+ (CD10+) and B-cell Lymphoma 6+ (BCL6+) markers, constitutes around half of all DLBCL cases. While the GCB subtype generally exhibits a better prognosis compared to the activated B-cell (ABC-) subtype, the high genetic heterogeneity within DLBCL frequently leads to unpredictable clinical outcomes [7].

Cancer arises from gene mutations and a microenvironment that drives aberrant proliferation and immune escape [8]. A significant genetic alteration associated with DLBCL is the chromosomal translocation involving the BCL6 gene, present in approximately 50% of cases [9]. BCL6, located on chromosome 3q27, encodes a 95 kDa transcriptional repressor essential for the formation of germinal centers in B-cell follicles during antigenic stimulation [10]. Structurally, the BCL6 protein contains three functional domains that contribute to DLBCL pathogenesis by regulating B-cell activation, differentiation, cell cycle arrest, and apoptosis [11,12]. Dysregulated BCL6 expression not only drives DLBCL progression but has also been implicated in various other malignancies. Elevated BCL6 levels have been detected in acute myeloid leukemia [13], glioma [14], ovarian cancer [15], non-small cell lung cancer [16,17], gastric cancer [18], and breast cancer [19,20], underscoring its role as a broader oncogenic factor.

Current multi-modal and precise combination therapies represent a highly effective strategy for personalized oncology [21]. Proteolysis-Targeting Chimeras (PROTAC) technology has shown significant potential in the targeted degradation of disease-related proteins [22], including BCL6. Several BCL6-targeting PROTAC degraders have been developed, demonstrating promising preclinical results [23]. For instance, BI-3802 promotes BCL6 polymerization and facilitates its interaction with the SIAH1 ubiquitin ligase, resulting in BCL6 ubiquitination and subsequent proteasomal degradation [24]. Although both BI-3802 and its analog BI-3812 effectively inhibit BCL6, allowing the reactivation of BCL6-repressed tumor suppressor genes, their limited bioavailability constrains their therapeutic potential for patients with BCL6-positive DLBCL [24]. Another compound, CCT373566, has been shown to induce BCL6 degradation in DLBCL cell lines; however, despite this degradation, it did not achieve sufficient antiproliferative effects, even in an extended 16-day proliferation study [25].

In the present study, to address the need for effective small-molecule degraders targeting BCL6 in GCB-DLBCL cells, we initially explored the expression in DLBCL compared with normal and other leukemia cells using bioinformatic analysis, validated by western blot in the different cells. We evaluated DZ-865B, a promising compound capable of inducing BCL6 degradation from our previous reports [26]. Unlike traditional cereblon-based PROTACs, DZ-865B was designed in reference to the small-molecule-induced BCL6 polymerization mechanism reported for BI-3802, which promotes SIAH1-mediated proteasomal degradation through an E3 ligase-independent of CRBN [24]. Targeting the transcriptional repressor BCL6, a key driver in DLBCL, this study aimed to identify and evaluate a novel PROTAC-based small molecule for its ability to degrade BCL6 and inhibit DLBCL growth, thereby laying the groundwork for preclinical development.

## 2 Materials and Methods

### 2.1 Experimental Design and Rationale

This study is an *in vitro* experimental investigation. This design was employed to eliminate interference from complex biological systems within a controlled culture environment, thereby enabling direct and precise assessment of DZ-865B’s effects on DLBCL cells.

### 2.2 Cell Culture

This study selected human diffuse large B-cell lymphoma cell lines OCI-LY-1 and SU-DHL-4, both of which are well-established models of the germinal center B-cell-like (GCB) subtype. This selection aimed to test DZ-865B in models most representative of DLBCL disease heterogeneity. Therefore, we selected the OCI-LY-1 and SU-DHL-4 cell lines, both of which are widely recognized and used in the field as models of the GCB subtype [27]. The human diffuse large B-cell lymphoma cells OCI-LY-1 and SU-DHL-4 cell lines were purchased from Zhejiang Mason Cell Technology Co. (Hangzhou, China). Gastric cancer cell lines MKN-45, HGC-27, and AGS were purchased from Wuhan Prolife Technology Co. (Wuhan, China). The human triple-negative breast cancer cell lines BT-549, MDA-MB-231, and BT-20 were purchased from the American Type Culture Collection (ATCC, Manassas, VA, USA). The non-small cell lung cancer (NSCLC) cell lines A549, NCI-H441, and NCI-H1299 were all purchased from the ATCC. The human normal bronchial epithelial cell line BEAS-2B, the human normal gastric mucosal epithelial cell line GES-1, and the normal renal tubular epithelial cell line HK-2 were all purchased from ATCC. All cell lines used in this study were authenticated by short tandem repeat (STR) profiling and confirmed to be free of mycoplasma contamination.

All cell lines were cultured under standard conditions (37°C, 5% CO_2_) in their respective recommended media, including RPMI-1640 (Cat. No. 11875093, Gibco™, Thermo Fisher Scientific, Waltham, MA, USA), DMEM (Cat. No. 11995065, Gibco™, Thermo Fisher Scientific, Waltham, MA, USA), or F-12K (Cat. No. 21127022, Gibco™, Thermo Fisher Scientific, Waltham, MA, USA), as appropriate. The media were supplemented with 10% fetal bovine serum (Cat. No. A5256701, Gibco™, Thermo Fisher Scientific, Waltham, MA, USA) and 1% penicillin-streptomycin (100×, Cat. No. 15140122, Gibco™, Thermo Fisher Scientific, Waltham, MA, USA).

All experiments in this study utilized cells maintained in a stable, healthy passage state. To control for potential confounding factors, experiments within the same group employed cells with identical passage numbers, identical seeding densities, and identical culture durations.

### 2.3 Cell Treatment and Drug Exposure

OCI-LY1 and SU-DHL-4 cells were treated with the BCL6 degrader BI-3802 or the experimental compound DZ-865B at a final concentration of 10 μm. Control groups received vehicle (DMSO) at equivalent volumes. Cells were incubated with compounds at 37°C under 5% CO_2_. All treatments were performed in three independent biological replicates. The proteasome inhibitor MG-132 (Cat. No. S42096, Yuanye, Shanghai, China) dissolved in DMSO was applied to OCI-LY1 and SU-DHL-4 cells at a final concentration of 300 nm for 2 h followed by treatment with DZ-865B for 48 h.

### 2.4 Western Blotting

Whole-cell proteins were extracted from the DLBCL (OCI-LY1 and SU-DHL-4), gastric cancer (MKN-45, HGC-27, and AGS), breast cancer (BT-549, MDA-MB-231, and BT-20), and NSCLC (A549, NCI-H441, and NCI-H1299) cell lines using the Whole Protein Extraction Kit (Cat. No. KGB5303-100, Keygen Biotech, Nanjing, China). After sonication, the samples were centrifuged (model 5425 R, Eppendorf SE, Hamburg, Germany) at 12,000 rpm for 15 min at 4°C and the total protein concentration of the samples was determined using the Micro BCA™ Protein Assay Kit (Cat. No. 23235, Thermo Fisher Scientific, Waltham, MA, USA). Protein samples were boiled for 10 min, and equal amounts (20 μg per lane) were loaded onto SDS-PAGE gels for electrophoresis. Protein blots were transferred to NC membranes (Cat. No. 66485, PALL, Port Washington, NY, USA) and incubated in blocking buffer containing 5% BSA (Albumin Bovine V, Cat. No. A6020, Biotopped, Beijing, China) for 1 h at room temperature, followed by incubation with appropriate primary and secondary antibodies (BCL6, Cat. No. 14895S, Cell Signaling Technology, Danvers, MA, USA; c-Myc, Cat. No. 18583, Cell Signaling Technology, Danvers, MA, USA; Cleaved Caspase-3, Cat. No. 9664, Cell Signaling Technology, Danvers, MA, USA; Bcl-xL, Cat. No. 2764, Cell Signaling Technology, Danvers, MA, USA; BAX, Cat. No. 60267-1-Ig, Proteintech Group, Wuhan, China; GAPDH, Cat. No. YM3029, Immunoway, San Jose, CA, USA; Beta Actin, Cat. No. 66009-1-Ig, Proteintech Group, Wuhan, China; Goat anti-mouse IgG secondary antibody, Cat. No. A23910, Abbkine, Wuhan, China; Goat anti-rabbit IgG secondary antibody, Cat. No. A23920, Abbkine, Wuhan, China). Subsequently, protein expression signals were detected using an Odyssey infrared two-color laser scanning imaging system (model 9120, Gene Company Limited, Hong Kong, China). Gray scale values were identified using ImageJ software (v1.53t, National Institutes of Health, Bethesda, MD, USA). All experiments were performed with three independent biological replicates.

### 2.5 Immunofluorescence

OCI-LY1 and SU-DHL-4 cells treated with 2.5, 5, or 10 μm of the DZ-865B in culture medium were fixed on ice using 4% paraformaldehyde (Cat. No. DF0130, Leagene, Beijing, China), permeabilized with 0.3% TritonX-100 (Cat. No. A110694, Sangon Biotech, Shanghai, China), and closed at room temperature with 1% BSA, followed by incubation with appropriate primary and secondary antibodies (BCL6, Cat. No. 14895S, Cell Signaling Technology, Danvers, MA, USA; Fluorescein (FITC)–conjugated Goat Anti-Rabbit IgG(H+L), Cat. No. SA00003-2, Proteintech Group, Wuhan, China). DAPI (DAPI dihydrochloride, Cat. No. C0065, Solarbio, Beijing, China) was added for nuclear staining and pictures were taken using a NIKON laser confocal microscope (model A1R HD25, Nikon Instruments Inc., Melville, NY, USA) in confocal specialized petri dishes (Cat. No. BDD011035, Biofil, Guangzhou, China).

### 2.6 Reverse Transcription Quantitative PCR (RT-qPCR)

OCI-LY1 and SU-DHL-4 cells were lysed by the E.Z.N.A Total RNA Kit (Omega Bio-tek, Norcross, GA, USA) and whole-cell RNA was extracted, and the concentration and quality of total RNA were measured using a NanoDrop 2000 (Thermo Fisher Scientific, Waltham, MA, USA). The proposed RNA was reverse transcribed and quantified by RT-qPCR. RT-qPCR instrument (Bio-Rad, Hercules, CA, USA) was used to analyze the expression levels of *BCL6*, *Bcl-xL*, *Bax*, *CXCR4* and *CDKN1A*. The gene expression levels of three independent experiments were calculated using the 2^−ΔΔCt^ method. The gene-specific primers are shown in the table below (Table 1):

**Table 1:** Primer sequences for RT-qPCR

Gene	Primer Sequences (5^′^-3^′^)
BCL6	F: GTCAGCAGCCTCCTCTTCTCC
	R: CGTGCCTCTTCTGGGATTGTTTC
Bcl-Xl	F: GAGAGCGTTCAGTGATCTAACATCC
	R: AGAACCACACCAGCCACAGTC
Bax	F: TTTCTGACGGCAACTTCAACTGG
	R: GATGGTGAGTGAGGCGGTGAG
CXCR4	F: ATTGTCATCCTGTCCTGCTATTGC
	R: AATGTCCACCTCGCTTTCCTTTG
CDKN1A	F: GTCACCGAGACACCACTGGAG
	R: AGCGAGGCACAAGGGTACAAG
GAPDH	F: GAAGGTGAAGGTCGGAGTC
	R: GAAGATGGTGATGGGATTTC

### 2.7 Soft Agar Cloning Assay

1.2% lower gel (Cat. No. A8190, Solarbio, Beijing, China) and 0.5% upper gel (Cat. No. A8190, Solarbio, Beijing, China) were prepared and the OCI-LY1 and SU-DHL-4 cells were inoculated on soft agar and given 2.5, 5, or 10 μm of the DZ-865B for incubation after 21 days of incubation, the cells were observed under an inverted microscope (model Axio Observer 7, Carl Zeiss AG, Jena, Germany) and photographed.

### 2.8 Cell Proliferation Assay

BEAS-2B, GES-1, and HK-2 cells were seeded in 96-well plates at an appropriate density of 3000 cells per well. After 72 h of treatment with serially diluted concentrations of the compound DZ-865B (ranging from 0.625 to 10 μm), cell proliferation was assessed using the methyl thiazolyl tetrazolium (MTT) assay kit (Cat. No. KGA9301-500, Keygen Biotech, Nanjing, China), 50 μl of 1× MTT reagent was added to each well, followed by incubation at 37°C for 4 h. After carefully removing the supernatant, 150 μl of dimethyl sulfoxide (DMSO) was added to each well. The plate was placed on an orbital shaker and agitated for 10 min to ensure thorough mixing, and the optical density was measured at 490 nm using an auto-mated spectrophotometer (model Multiskan GO, Thermo Fisher Scientific, Waltham, MA, USA). The IC_50_ values were calculated using GraphPad Prism software (v9.5, GraphPad Software, Boston, MA, USA).

### 2.9 EDU Cell Proliferation Assay

OCI-LY1 and SU-DHL-4 cells were seeded in 60-mm dishes at a density of 1–2 × 10^4^ cells per dish and treated with DZ-865B (0, 2.5, 5, 10 μm) for 48 h. Cells were pulsed with 10 μm EdU for 2 h at 37°C, fixed with 3.7% formaldehyde, and permeabilized with 0.5% Triton X-100. EdU incorporation was detected using the EdU Method Cell Proliferation Imaging Analysis Kit (green fluorescence) (Cat. No. KTA2030, Abbkine, Wuhan, China) according to the manufacturer’s protocol. Nuclei were counterstained with Hoechst 33342. Images were acquired using a Nikon laser confocal microscope (model A1R HD25, Nikon Instruments Inc., Melville, NY, USA).

### 2.10 Immunohistochemical Staining

The Human Protein Atlas (HPA, http://www.proteinatlas.org/) is a comprehensive online resource designed to provide detailed information on human protein expression and localization. The database integrates a wide range of immunohistochemistry, cellular immunohistochemistry, proteomics, and histology data, providing valuable information for studying the expression patterns of human proteins at the tissue and cellular levels. Immunohistochemical staining images of four tumor groups and paraneoplastic tissues were downloaded from the Human Protein Atlas for analysis.

### 2.11 Bioinformatics Analysis of BCL6

The expression level of BCL6 was analyzed using Gene Expression Omnibus (GEO, https//www.ncbinlm.nih.gov/geo/) based on the GSE32018 dataset, with 13 cases in the normal tissue group (including lymph nodes and reactive tonsils), 17 cases in the group of patients with chronic lymphocytic leukemia, 22 cases in the group of patients with diffuse large B-cell lymphoma, and 75 cases in the group of patients with other lymphomas. The group of 75 patients included patients with follicular lymphoma, pocket cell lymphoma, marginal zone lymphoma-MALT type, and node-marginal zone lymphoma.

### 2.12 Quantum Calculation

The highest occupied molecular orbital (HOMO) and lowest unoccupied molecular orbital (LUMO) energies of DZ-865B were calculated using the Python-based Simulations of Chemistry Framework (PySCF, v2.3.0). For this study, the B3LYP (Becke, three-parameter, Lee-Yang-Parr) exchange-correlation functional was employed. The 6-311G basis set was chosen to provide a balance between computational efficiency and accuracy for the HOMO and LUMO calculations.

The structure of DZ-865B was first optimized at the B3LYP/6-311G level, ensuring that it reached a stable conformation with minimum energy. Subsequently, the HOMO and LUMO energies were calculated based on the optimized structure. Presentation of HOMO and LUMO was conducted by Avogadro software (v1.2.0) [28].

### 2.13 Protein Docking Assays

The coordinate files for SIAH1 and BCL6 were processed with UCSF Chimera (v1.15) to remove all heteroatoms (water molecules, ligands, and ions). Any missing side chains or loops were built using the Modeller module within Chimera, guided by the alignment to homologous regions when available. Protonation states were assigned at pH 7.4 using PROPKA within PDB2PQR (v3.5.1), and all histidine residues were manually checked to ensure correct tautomeric forms. The resulting PQR files were converted back to PDB format and energy-minimized in implicit solvent for 1000 steps of steepest-descent followed by 2000 steps of conjugate-gradient refinement using CHARMM-GUI’s (v3.8, July 2022) [29] Quick MD setup (CHARMM36m force field), until the root-mean-square deviation (RMSD) change between iterations was less than 0.02 Å. Finally, all atom names and chain identifiers were standardized to be compatible with ZDOCK3.

The prepared SIAH1 structure was designated as the “receptor” and BCL6 as the “ligand”, and rigid-body docking was performed using ZDOCK 3.0.2 on a Linux workstation (Intel Xeon E5-2650 v4, 2.20 GHz; 64 GB RAM) with default parameters except where noted: ligand orientations were sampled on a uniform angular grid with 6° increments (10,800 poses), and both proteins were mapped onto a 1.2 Å 3D cubic grid for shape-complementarity scoring via FFT. Electrostatic and desolvation contributions were assessed using the standard ZDOCK scoring function (shape complementarity, electrostatics, and atomic contact energy) with a dielectric constant of 4.0 to approximate intra-protein screening, and the top 2000 poses were retained. These were rescored by ZRANK v2.1 (weighted van der Waals, electrostatics, and desolvation energies), and the 200 highest-ranking models were subjected to RDOCK v2.0 refinement, during which interfacial side-chain conformations were optimized by a 3000-step Monte Carlo minimization followed by an empirical free-energy evaluation.

### 2.14 Molecular Docking

The 2D chemical structure of DZ-865B was prepared using ChemDraw (v23.0.0, PerkinElmer, Waltham, MA, USA) and converted to a three-dimensional (3D) model using OpenBabel (v2.4.1). Geometry optimization was performed by the MMFF94 force field for 500 steps of steepest-descent minimization. The resulting structure was imported into AutoDockTools 1.5.6, where all non-polar hydrogen atoms were merged, and Gasteiger partial charges were assigned. Rotatable bonds were detected automatically, and torsional degrees of freedom were set following default AutoDock conventions. The ligand was then saved in PDBQT format with defined rotatable bonds for docking.

We selected the highest-scoring SIAH1-BCL6 complex from the ZDOCK3 pipeline as the receptor template. Using UCSF Chimera (v1.15), all crystallographic water molecules and heteroatoms were removed, and any missing side chains were reconstructed via Modeller (integrated into Chimera) to restore complete backbone and side-chain geometry. Protonation states at pH 7.4 were assigned with PROPKA (via PDB2PQR), and the structure was energy-minimized (CHARMM36m) for 1000 steps of steepest-descent followed by 2000 steps of conjugate-gradient refinement (implicit solvent) to relieve steric clashes. The minimized complex was imported into AutoDockTools 1.5.6, polar hydrogens were added, and Kollman united-atom charges were assigned. The receptor was saved in PDBQT format with side chains kept rigid for docking.

Based on interface residues identified from the RDOCK-refined complex, a cubic grid box was defined to encompass the SIAH1–BCL6 interface pocket. The grid center was set at (X = 47.696, Y = 94.744, Z = 81.858) in the receptor’s coordinate frame, with dimensions of 72 × 76 × 68 grid points and a grid spacing of 0.375 Å. This volume accommodated the entire predicted binding groove plus a 5 Å buffer around key interface side chains. All the docking results were analyzed and presented using Ligplot+(v2.3).

### 2.15 Statistical Analysis

All data were analyzed using GraphPad Prism 8.0 software (GraphPad Software, Inc., San Diego, CA, USA). The data were represented as mean ± standard deviation. Prior to conducting parametric tests, all data underwent verification for normality of distribution (Shapiro-Wilk test) and homogeneity of variance (Levene’s test). Significant differences were identified through the use of a *t*-test and analysis of variance (ANOVA), followed by Tukey’s HSD test for post hoc multiple comparisons, with *p*-values less than 0.05 considered to be statistically significant. To control for potential confounding factors, this study preemptively controlled major sources of variation through experimental design. Each experiment was conducted in triplicate to ensure reproducibility.

## 3 Results

### 3.1 BCL6 Highly Expressed in DLBCL

**Figure 1:**
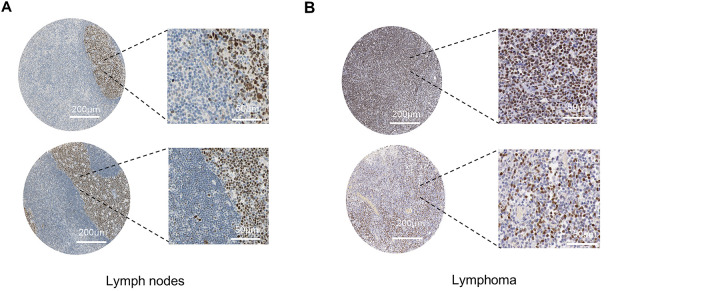

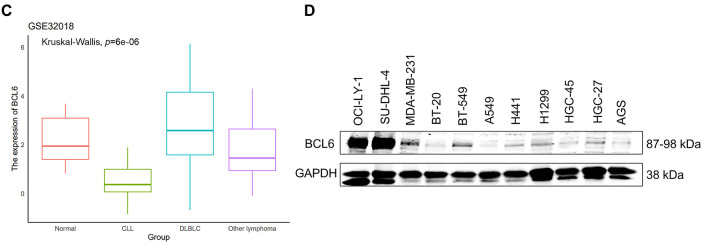
Elevated BCL6 Expression in Lymphoma. (**A**). Immunohistochemical staining of lymph node tissues from B-cell germinal centers, demonstrating medium to strong nuclear staining for BCL6. Scale bars: 200 and 50 μm. (**B**). Immunohistochemical staining of diffuse large B-cell lymphoma (DLBCL) tissue samples, showing robust nuclear BCL6 expression. Scale bars: 200 and 50 μm. (**C**). Comparative analysis of BCL6 expression across different patient groups, including normal tissues (Normal, n = 13), chronic lymphocytic leukemia (CLL, n = 17), diffuse large B-cell lymphoma (DLBCL, n = 22), and other lymphomas (Tumor, n = 75). Statistical significance was assessed using Kruskal-Wallis analysis. (**D**). Western blot analysis of BCL6 protein levels in various tumor cell lines, confirming higher expression in DLBCL cell lines compared to other tumor types

In studies of DLBCL, aberrant expression of the BCL6 gene has been identified as a critical factor in disease development. To explore BCL6 expression in DLBCL, we first analyzed lymph node tissues from B-cell germinal centers and non-germinal centers using immunohistochemistry from the Human Protein Atlas (HPA) database. Our results revealed higher BCL6 expression in germinal centers, evidenced by medium to strong nuclear staining (Fig. 1A). Immunohistochemical analysis of DLBCL tissue samples confirmed robust nuclear staining of BCL6 in lymphoma cells (Fig. 1B). Furthermore, differential gene expression analysis using the GEO database showed that BCL6 was significantly upregulated in DLBCL patient samples (*p* = 6.0e−6) (Fig. 1C). Western blotting of DLBCL cell lines, specifically OCI-LY-1 and SU-DHL-4, also indicated markedly higher levels of BCL6 protein compared to other tumor cell lines (Fig. 1D). These findings collectively suggest that BCL6 is highly and specifically expressed in DLBCL, underscoring its potential role as a therapeutic target.

### 3.2 Identification of DZ-865B as a Novel BCL6 Degradation

**Figure 2:**
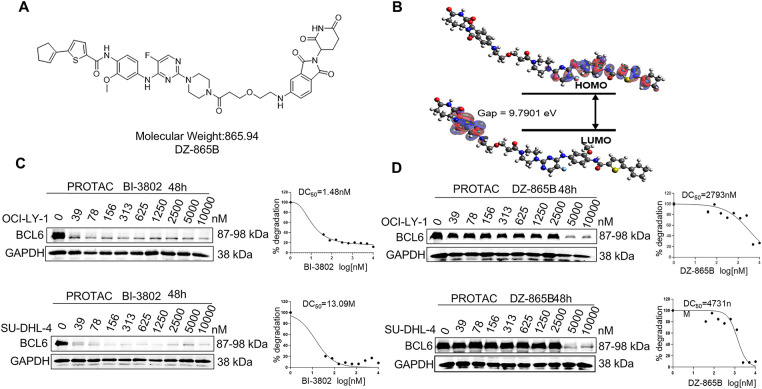
Identification of DZ-865B as a Novel BCL6 Degrader. (**A**). Chemical structure of DZ-865B utilized in this study. (**B**). Highest occupied molecular orbital (HOMO) and lowest unoccupied molecular orbital (LUMO) of DZ-865B, calculated using PySCF with the B3LYP/6-311G method. (**C**). Western blot analysis of BCL6 expression in DLBCL cell lines (OCI-LY-1 and SU-DHL-4) following treatment with the known BCL6 degrader BI-3802, used as a positive control. (**D**). Western blot analysis of BCL6 expression in DLBCL cell lines (OCI-LY-1 and SU-DHL-4) following treatment with DZ-865B, demonstrating its efficacy as a BCL6 degrader

To degrade the BCL6 *in vivo*, we designed an emerging PROTAC molecules, and the chemical structure of DZ-865B, evaluated in this study, is shown in Fig. 2A. To understand its electronic properties, we calculated the highest occupied molecular orbital (HOMO) and lowest unoccupied molecular orbital (LUMO) of DZ-865B using PySCF with the B3LYP/6-311G method (Fig. 2B). These calculations provide insights into the electronic distribution of DZ-865B, which may influence its interaction with target proteins. To assess the BCL6-degrading potential of DZ-865B, we performed Western blot analyses on two DLBCL cell lines, OCI-LY-1 and SU-DHL-4. Initially, we treated these cells with BI-3802, a known BCL6 degrader, as a positive control. As expected, BI-3802 treatment led to a marked decrease in BCL6 protein levels in both cell lines, consistent with BCL6 degradation (Fig. 2C). Subsequently, we treated OCI-LY-1 and SU-DHL-4 cells with DZ-865B and evaluated BCL6 expression via Western blotting. Similar to BI-3802, DZ-865B treatment significantly reduced BCL6 protein levels in a concentration-dependent manner in both cell lines, consistent with its role as a BCL6-targeting agent (Fig. 2D). These findings demonstrate that DZ-865B functions as a novel BCL6 degrader and holds potential as a therapeutic agent for DLBCL by facilitating the degradation of BCL6 in cancer cells.

**Figure 3:**
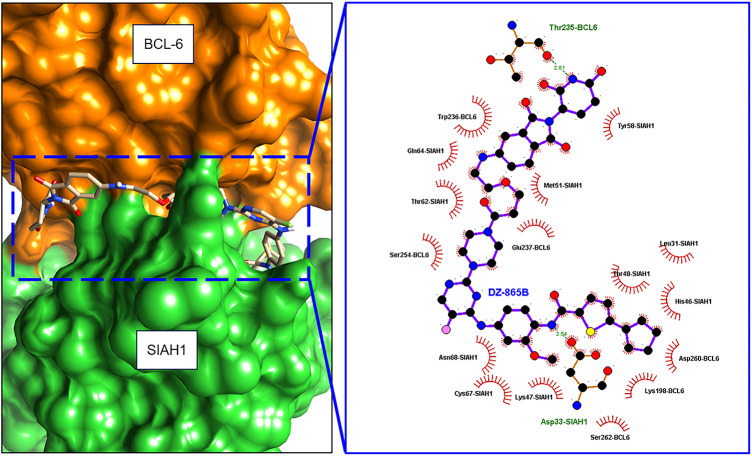
Molecular docking of DZ-865B at the SIAH1–BCL6 interface. Orange section was BCL-6 protein, and green section was SIAH1 protein. Planar molecular interaction model was presented using Ligplot+ software. Hydrogen bond was labeled with a dashed line

To evaluate whether DZ-865B could stably occupy the interface between SIAH1 and BCL6, the highest-ranking ZDOCK3 model of the SIAH1–BCL6 complex was used as receptor and DZ-865B was docked using AutoDock4.2 (Fig. 3). The top consensus conformation exhibits a predicted binding free energy of −11.6 kcal mol^−1^ and locates the ligand squarely within a contiguous pocket formed by residues contributed by both SIAH1 and BCL6. In this pose, DZ-865B forms two critical hydrogen bonds: its central amide engages the side chain of Thr 235 in BCL6 (2.81 Å) and the sulfonamide moiety hydrogen-bonds to Asp 33 in SIAH1 (2.54 Å). These polar interactions are reinforced by a network of hydrophobic contacts involving SIAH1 residues Met 51, Thr 62, Gln 64, Tyr 58, Leu 31, Thr 48, His 46, Cys 67 and Lys 47, as well as BCL6 residues Trp 236, Ser 254, Glu 237, Asp 260, Lys 198 and Ser 262. Notably, the aromatic ring of Trp 236 (BCL6) stacks against the bent heterocycle of DZ-865B, further stabilizing the complex. Upon minimization in CHARMM36m (implicit solvent), the selected docked complex retains its hydrogen-bond geometry and experiences an energy drop of 23 kcal mol^−1^, consistent with a favourable binding mode. Collectively, these data indicate that DZ-865B bridges key interfacial residues of SIAH1 and BCL6, providing a structural basis for the disruption of their protein-protein interaction.

### 3.3 DZ-865B Induces Concentration- and Time-Dependent Degradation of BCL6 in DLBCL Cell Lines

**Figure 4:**
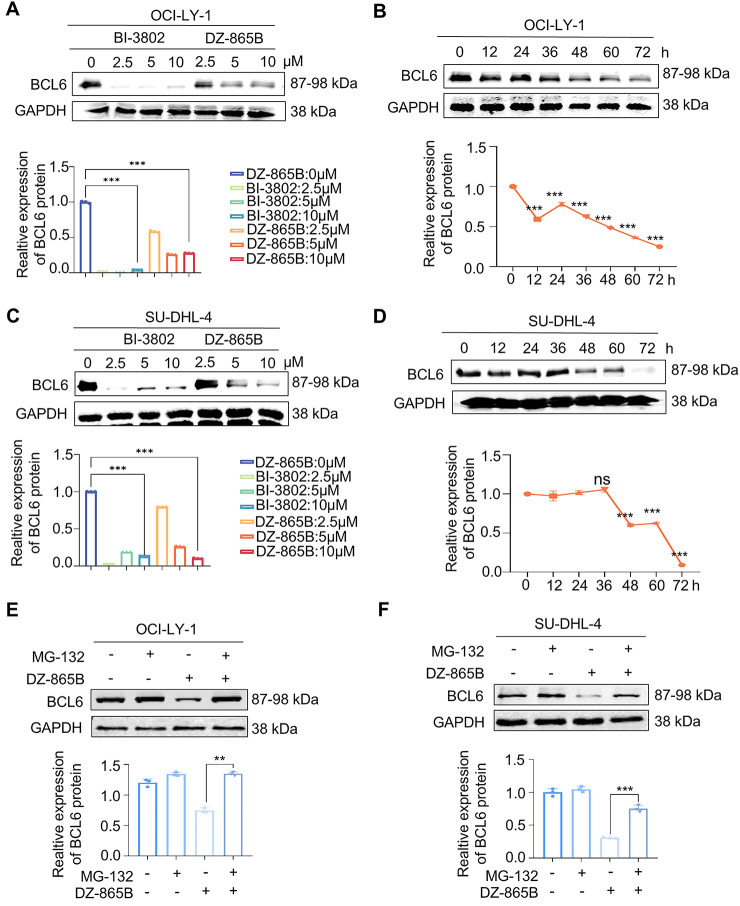

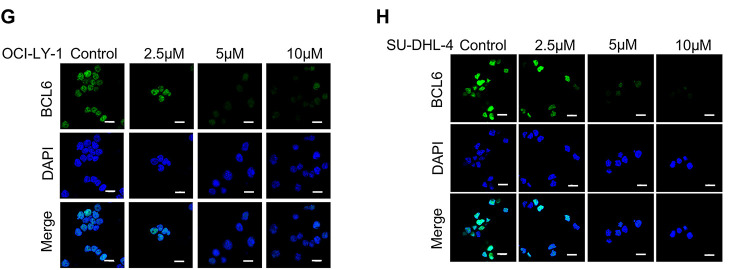
Concentration- and time-dependent degradation of BCL6 by DZ-865B in DLBCL cell lines. (**A**,**C**) Western blot analysis showing the concentration-dependent degradation of BCL6 in DLBCL cell lines OCI-LY-1 and SU-DHL-4 following treatment with varying concentrations of DZ-865B. Quantitative analysis of the Western blot bands, presented as histograms. Significant degradation of BCL6 is observed compared to control (***p < 0.001). (**B**,**D**) Western blot analysis depicting the time-dependent degradation of BCL6 in OCI-LY-1 and SU-DHL-4 cell lines after treatment with DZ-865B at specific time intervals. Quantitative analysis of the Western blot bands over time, presented as line graphs, with a significant reduction in BCL6 levels compared to control (***p < 0.001, ns, not significant). (**E**,**F**) OCI-LY-1 and SU-DHL-4 cells were pretreated with 300 nm MG-132 for 2 h, followed by treatment with 10 μm DZ-865B for 48 h. BCL6 protein levels were then analyzed by Western blot. Statistical significance was assessed using two-tailed unpaired Student’s *t*-test (**p < 0.01, ***p < 0.001). (**G**,**H**) Immunofluorescence images demonstrating the disruption of BCL6 colocalization in DLBCL cells upon treatment with DZ-865B, the control group did not receive DZ-865B treatment. Scale bar: 10 μm

To evaluate the efficacy of DZ-865B in targeting BCL6, we performed concentration- and time-dependent assays in DLBCL cell lines OCI-LY-1 and SU-DHL-4. Western blot analyses demonstrated a dose- and time-dependent reduction in BCL6 protein levels upon treatment with increasing concentrations of DZ-865B in both cell lines (Fig. 4A,C). Quantitative analysis of these Western blot results confirmed a significant decrease in BCL6 expression, with statistical analysis indicating high significance compared to controls. Next, we examined the time-dependent effects of DZ-865B on BCL6 degradation. Western blot analysis revealed a progressive decrease in BCL6 protein levels in both OCI-LY-1 and SU-DHL-4 cell lines over time following DZ-865B treatment (Fig. 4B,D). Quantitative assessment of these time-course data, shown as line graphs, indicated a significant reduction in BCL6 expression at all time points compared to the control group.

To investigate the mechanism of DZ-865B-induced BCL6 degradation, we assessed whether the proteasome inhibitor MG-132 could rescue this effect. Indeed, pretreatment with MG-132 partially restored BCL6 protein levels in both OCI-LY-1 and SU-DHL-4 cells. It indicates that DZ-865B degrades BCL6 protein through the ubiquitin-proteasome system under the PROTACs mechanism (Fig. 4E,F).

To further explore the effects of DZ-865B on BCL6 localization, we conducted immunofluorescence staining. Treatment with DZ-865B changed BCL6 subcellular colocalization in DLBCL cells, as indicated by the altered nuclear staining pattern. These changes in BCL6 localization were observed in both cell lines, further supporting the degradation and functional modulation of BCL6 by DZ-865B (Fig. 4G,H).

### 3.4 DZ-865B Regulation of BCL6 Downstream Gene Expression

**Figure 5:**
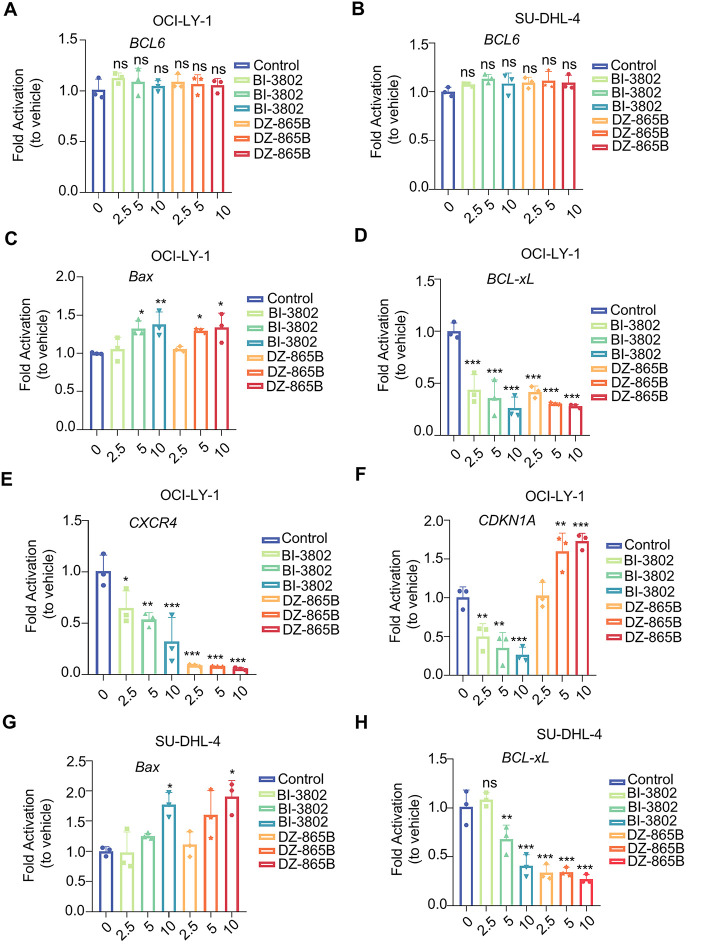

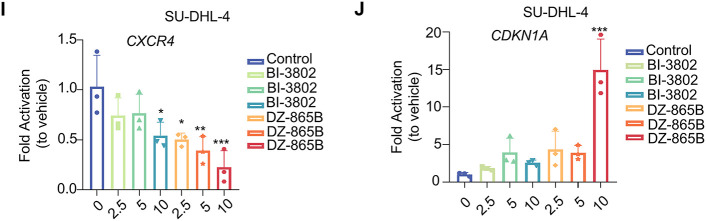
DZ-865B modulates expression of BCL6 downstream genes in DLBCL Cell Lines. (**A**,**B**) RT-qPCR analysis showing that DZ-865B treatment does not significantly affect BCL6 mRNA levels in OCI-LY-1 and SU-DHL-4 cell lines, indicating that DZ-865B regulates BCL6 at the protein level rather than through transcriptional downregulation (ns, not significant). (**C**–**F**) Relative mRNA expression of BCL6 target genes in OCI-LY-1 cells after treatment with DZ-865B. Data reveal a significant reduction in the mRNA levels of BCL6-regulated genes, with statistical significance indicated by *p < 0.05, **p < 0.01, and ***p < 0.001 compared to control levels. (**G**–**J**) Relative mRNA expression of BCL6 target genes in SU-DHL-4 cells following exposure to DZ-865B. Similar to OCI-LY-1 cells, treatment with DZ-865B leads to significant downregulation of specific BCL6 target genes, with statistical significance marked by *p < 0.05, **p < 0.01, and ***p < 0.001 vs. control, ns, not significant

BCL6 is a crucial transcriptional repressor involved in the regulation of various cellular processes, including proliferation, differentiation, and apoptosis. It exerts its effects by modulating the expression of downstream target genes. To investigate the regulatory effects of DZ-865B on BCL6 and its target genes, we treated BCL6-overexpressing DLBCL cell lines (OCI-LY-1 and SU-DHL-4) with increasing concentrations of DZ-865B (0, 2.5, 5, and 10 μm) for 48 h. Reverse Transcription Quantitative PCR (RT-qPCR) analysis revealed that DZ-865B had no significant effect on BCL6 mRNA levels, indicating that the compound does not alter BCL6 at the transcriptional level (Fig. 5A,B).

However, treatment with DZ-865B significantly modulated the expression of BCL6-regulated downstream genes in a concentration-dependent manner. Specifically, DZ-865B upregulated the expression of pro-apoptotic genes, such as *Bax* and *CDKN1A*, while downregulating anti-apoptotic and cell survival-related genes, including *Bcl-xL* and *CXCR4*, in both OCI-LY-1 (Fig. 5C–F) and SU-DHL-4 cell lines (Fig. 5G–J). This effect was more pronounced than that of the positive control compound, BI-3802, at the same concentration. These findings suggest that DZ-865B exerts a stronger regulatory impact on the transcriptional network controlled by BCL6, highlighting its potential as a therapeutic agent targeting BCL6-mediated pathways in DLBCL.

### 3.5 DZ-865B Inhibits DLBCL Cell Proliferation In Vitro

**Figure 6:**
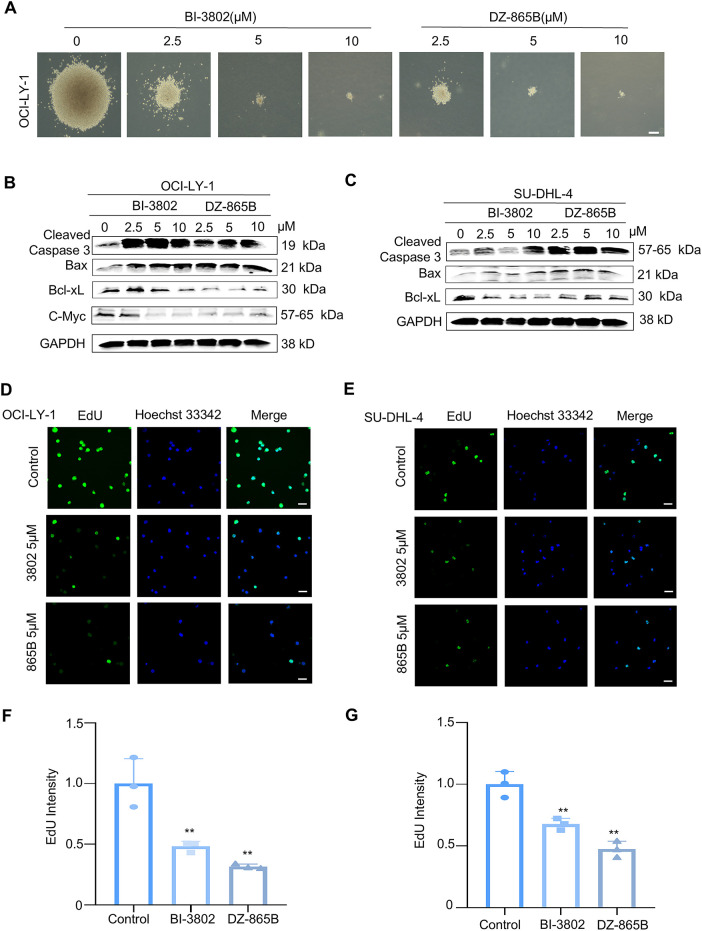
DZ-865B inhibits proliferation of DLBCL Cells *In Vitro*. (**A**). Soft agar colony formation assay of DLBCL cell line OCI-LY-1 after 21 days of treatment with various concentrations of DZ-865B, showing a dose-dependent decrease in colony formation. Scale bar: 10 μm. (**B**). Western blot analysis of OCI-LY-1 cells treated with BI-3802 and DZ-865B for 48 h, examining the expression levels of apoptosis and proliferation-related proteins, including Cleaved Caspase-3, Bax, Bcl-xL, and C-Myc, in response to increasing concentrations of each compound. (**C**). Western blot analysis of SU-DHL-4 cells treated with BI-3802 and DZ-865B for 48 h, assessing the expression of Cleaved Caspase-3, Bax, and Bcl-xL to evaluate apoptotic and anti-apoptotic protein modulation in a concentration-dependent manner. (**D**,**E**). EdU incorporation assay to assess DNA synthesis in OCI-LY-1 and SU-DHL-4 cell lines treated with 5 μm DZ-865B for 48 h. Immunofluorescence images show reduced EdU-positive cells after DZ-865B treatment, indicating decreased DNA synthesis and proliferation. Scale bar: 10 μm. (**F**,**G**). Quantitative analysis of EdU fluorescence intensity, measured as integrated density using ImageJ software. The control group did not receive DZ-865B treatment. Results indicate a significant reduction in DNA synthesis in DZ-865B-treated groups compared to control groups (**p < 0.01 vs. control)

BCL6 is essential for the regulation of B-cell development and function. In certain cases of DLBCL, BCL6 expression is abnormally elevated, disrupting the balance of B-cell proliferation and promoting the uncontrolled growth of DLBCL cells. In this study, DZ-865B significantly inhibited the formation of tumor cell colonies, as demonstrated by the soft agar assay (Fig. 6A). To evaluate the selective toxicity of DZ-865B, we assessed its cytotoxicity against normal cell lines with low BCL6 expression (including BEAS-2B, GES-1, and HK-2) using the MTT assay. The results demonstrated that even at a high concentration of 10 μm, DZ-865B did not significantly inhibit the viability of these normal cells, indicating its selective inhibitory profile and favorable safety properties (Table A1). Additionally, DZ-865B treatment resulted in a marked downregulation of C-Myc, a protein associated with cell proliferation, indicating that DZ-865B suppresses the growth of OCI-LY-1 cells (Fig. 6B,C).

To further explore the impact of DZ-865B on apoptosis-related proteins, we analyzed key markers of apoptosis in both OCI-LY-1 and SU-DHL-4 cell lines. In OCI-LY-1 cells, DZ-865B treatment led to increased levels of Cleaved Caspase-3, a critical executioner of apoptosis, along with Bax, a pro-apoptotic protein, and decreased expression of Bcl-xL, an anti-apoptotic protein, in a dose-dependent manner. Similarly, in SU-DHL-4 cells, DZ-865B upregulated Cleaved Caspase-3 and Bax and downregulated Bcl-xL, indicating enhanced pro-apoptotic signaling. Notably, DZ-865B showed a more pronounced effect on these apoptotic markers compared to the positive control compound, BI-3802, at equivalent concentrations (Fig. 6B,C).

The EdU incorporation assay was employed to assess the impact of DZ-865B on DNA synthesis, a key indicator of cell proliferation [24]. Following 48 h of treatment with 5 μm DZ-865B, both OCI-LY-1 and SU-DHL-4 cells exhibited a significant reduction in EdU-positive cells (Fig. 6D,E). Quantitative analysis of EdU fluorescence intensity confirmed a significant decrease in DNA synthesis in the DZ-865B-treated groups compared to the control, further supporting the anti-proliferative effect of DZ-865B in DLBCL cells (Fig. 6F,G).

## 4 Discussion

In recent years, the advent of proteolysis-targeting chimera (PROTAC) technology has opened new avenues for targeting proteins previously considered undruggable [30,31]. A similar strategy has been applied for Voltage-Gated Sodium Channel (VGSC) [32]. PROTACs induce the ubiquitination and subsequent proteasomal degradation of specific proteins, thereby directly modulating their function [33]. Efforts to develop PROTACs targeting the BTB domain of BCL6 have shown promise, yet challenges remain. For instance, targeting the side groove of the BCL6 BTB domain to disrupt interactions with co-repressor proteins can lead to degradation of BCL6 aggregates, inadvertently exposing the pro-inflammatory effects of BCL6 deficiency [34]. Additionally, while BCL6-targeting PROTACs have achieved cellular concentrations sufficient for degradation, they have not yet demonstrated significant phenotypic responses in DLBCL, likely due to suboptimal pharmacokinetic and pharmacodynamic properties [25]. To date, no direct BCL6 degraders have received FDA approval, underscoring the need for further optimization of these compounds [23,35,36].

To address these challenges, we identified a novel small molecule degrader, DZ-865B, and investigated its efficacy in BCL6-overexpressing DLBCL cells. DZ-865B was found to degrade BCL6 in a concentration- and time-dependent manner, significantly reducing nuclear BCL6 levels in DLBCL cell lines (OCI-LY-1 and SU-DHL-4). Notably, while DZ-865B did not affect BCL6 mRNA expression, it modulated the transcriptional profile of BCL6-regulated genes, upregulating pro-apoptotic markers such as *Bax* and *CDKN1A* and downregulating anti-apoptotic and cell survival genes like *Bcl-xL* and *CXCR4*. These findings suggest that DZ-865B acts primarily by degrading BCL6 protein, which in turn disrupts downstream pathways essential for DLBCL cell survival.

Functionally, DZ-865B exhibited potent anti-proliferative effects in DLBCL cells at relatively low concentrations, as demonstrated by soft agar colony formation and EdU incorporation assays. DZ-865B treatment did not significantly inhibit the viability of normal cells. DZ-865B achieves targeted degradation of BCL6 through the ubiquitin-proteasome system, thereby more fundamentally disrupting the oncogenic signaling network driven by BCL6. Treatment with DZ-865B led to a reduction in C-Myc expression, a key regulator of cell proliferation, and increased levels of pro-apoptotic markers such as Cleaved Caspase-3 and Bax, while downregulating the anti-apoptotic protein Bcl-xL. These results confirm that DZ-865B not only inhibits DLBCL cell growth by directly degrading BCL6 through the ubiquitin-proteasome pathway, but also promotes apoptosis, indicating its potential as a therapeutic candidate for BCL6-driven malignancies. By specifically targeting the abnormal expression of BCL6, DZ-865B could help restore normal apoptotic processes, thereby limiting the uncontrolled proliferation of DLBCL cells.

This study demonstrates that DZ-865B serves as a valuable chemical tool and provides a promising lead compound for developing therapeutic strategies against BCL6-dependent DLBCL. Looking forward, it is noteworthy that DZ-865B does not incorporate a cereblon-binding motif and thus does not exploit the CRBN pathway commonly used by many heterobifunctional degraders. Instead, our docking results support a SIAH1-dependent degradation mechanism, similar to BI-3802 [24], subsequent research should focus on rational structural optimization based on its current framework to develop degraders with higher selectivity and superior *in vivo* performance. Furthermore, multi-omics analysis [37] and investigation of the signaling networks following BCL6 degradation will provide critical insights into its regulatory mechanisms and guide the development of combination therapeutic strategies. This direction aligns with the broader shift toward precision medicine in cancer treatment—where targeting the tumor immune microenvironment and underlying molecular mechanisms represents the future of cancer therapy [38]. The targeted protein degradation strategy, as exemplified by DZ-865B, holds promise for contributing more effective tools to the precision management system for lymphoma, thereby expanding the treatment options available in precision oncology. Previous studies have reported that BCL6 inhibitors can be utilized in combination with other targeted agents to overcome lymphoma heterogeneity. Future research will explore combination strategies between PROTAC degraders and additional therapeutic compounds to further improve treatment efficacy [39–41].

Although DZ-865B has limitations in terms of efficacy and drug properties, as a prototype compound, it successfully verified the feasibility of the PROTAC-mediated BCL6 degradation strategy. The current work not only provides a strong proof of concept but also points out a clear optimization direction for the subsequent development of a new generation of BCL6 degraders with better degradation efficiency and pharmacokinetic characteristics.

## 5 Conclusion

Our findings indicate that DZ-865B effectively targets BCL6 in DLBCL cells, leading to a marked inhibition of BCL6 expression and subsequent activation of apoptosis-related genes. This mechanism significantly reduces the proliferative capacity of DLBCL cells, suggesting that DZ-865B could serve as a potent therapeutic agent for BCL6-driven lymphomas. The specific downregulation of BCL6 and the induction of apoptosis-related markers emphasize DZ-865B’s potential role in overcoming the growth and survival advantages conferred by BCL6 overexpression in these tumors. Given the emergence of BCL6-targeting compounds DZ-865B, our investigation underscores the importance of continued exploration of BCL6 as a therapeutic target by specific BCL6 degradation, particularly in treatment-resistant DLBCL cases. DZ-865B serves as a critical proof-of-concept tool by demonstrating actionable degradation of BCL6. It not only validates the PROTAC-mediated BCL6 degradation strategy but also illuminates a clear path for the development of optimized successors with superior degradation efficiency and pharmacokinetic characteristics. These findings further highlight the need for precision oncology approaches that assess BCL6 expression and downstream pathway alterations in order to tailor targeted therapies effectively.

## Data Availability

The data that support the findings of this study are available from the Corresponding Authors, upon reasonable request.

## Appendix A

**Table A1:** Antiproliferative activities of representative compounds against cytotoxicities on normal cell line

Cell lines		Normal cell lines		
		BEAS-2B	GES-1	HK-2
**Compounds IC**_**50**_ **(mean ± SD μM)**	DZ-865B	>10	>10	>10
	BI-3802	>10	>10	>10
